# Stress-Induced Premature Senescence or Stress-Induced Senescence-Like Phenotype: One *In Vivo* Reality, Two Possible Definitions?

**DOI:** 10.1100/tsw.2002.100

**Published:** 2002-01-29

**Authors:** Olivier Toussaint, Patrick Dumont, Jose Remacle, Jean-Francois Dierick, Thierry Pascal, Christophe Frippiat, Joao Pedro Magalhaes, Stephanie Zdanov, Florence Chainiaux

**Affiliations:** University of Namur (FUNDP), Department of Biology, Research Unit of Cellular Biology (URBC), Namur, Belgium; Department of Pharmacology, Fox Chase Cancer Center, 7701 Burholme Ave., Philadelphia, PA 19111, USA

**Keywords:** senescence, oxidative stress, cell cycle, telomeres, TGF-β1, aging

## Abstract

No consensus exists so far on the definition of cellular senescence. The narrowest definition of senescence is irreversible growth arrest triggered by telomere shortening counting cell generations (definition 1). Other authors gave an enlarged functional definition encompassing any kind of irreversible arrest of proliferative cell types induced by damaging agents or cell cycle deregulations after overexpression of proto-oncogenes (definition 2). As stress increases, the proportion of cells in “stress-induced premature senescence-like phenotype” according to definition 1 or “stress-induced premature senescence,” according to definition 2, should increase when a culture reaches growth arrest, and the proportion of cells that reached telomere-dependent replicative senescence due to the end-replication problem should decrease. Stress-induced premature senescence-like phenotype and telomere-dependent replicatively senescent cells share basic similarities such as irreversible growth arrest and resistance to apoptosis, which may appear through different pathways. Irreversible growth arrest after exposure to oxidative stress and generation of DNA damage could be as efficient in avoiding immortalisation as “telomere-dependent” replicative senescence. Probabilities are higher that the senescent cells (according to definition 2) appearing *in vivo* are in stress-induced premature senescence rather than in telomere-dependent replicative senescence. Examples are given suggesting these cells affect *in vivo* tissue (patho)physiology and aging.

## INTRODUCTION

All biological systems have been exposed to stress since the dawn of biological time. Stress participates in natural selection and biological evolution. The immediate cellular response to stimulations (by growth factors and cytokines) and the immediate response to stress (like reactive oxygen species [ROS], ionizing radiations, osmotic stress, mechanical stress, hypoxia, heavy metals, and heat shocks) are drawing much interest. The long-term cellular response to stress, from several days to several weeks after the stress, also receives deserved attention. According to the net result of the balance between the damage generated by stress and the efficiency of the stress response, various cellular behaviors are expected. A theoretical framework was developed that helps explain the complex relationships between stress and senescence. It is worthwhile developing this theoretical framework before exploring these relationships.

## THEORETICAL FRAMEWORK: EFFECTS OF STRESS ON CELLULAR BEHAVIORS

First, constant ubiquitous mild stress due to conditions such as the basal concentrations of ROS do not immediately alter the stability of the cellular systems. Nevertheless, it represents weak variations of one or several biological parameters vs. time. When repair does not take place rapidly, some of the modifications may become irreversible and accumulate without immediate alteration of the system stability. Some cellular subsystems might work as compensatory mechanisms counteracting the irreversible errors accumulating in other cellular subsystems. When the level of damage reaches a threshold at which these compensatory mechanisms become, even transiently, overwhelmed, the system stability is lost[1]. (Far-from-equilibrium thermodynamic developments were published in[1].) Then the cell shifts to a new state of organisation. This new thermodynamic steady state is characterized by a higher level of damage and lower global biochemical activity. This process of shifting through a series of (possibly numerous) new steady states with time due to accumulation of damage is the manifestation of normal aging. When the level of intracellular damage increases and the global biochemical activity decreases too much, the cells die either by apoptosis or, if activation of apoptosis is no longer possible for any reason, by necrosis. This long process of damage accumulation could explain the “senescence” of nonproliferative cell types. Telomere shortening occurring discretely at each cell division in proliferative cells, in the absence of telomerase activity, can be seen as an example of damage that accumulates due to the lack of an efficient repair system and/or perfect DNA duplication mechanisms. We shall discuss later the order of occurrence of “senescence” of proliferative cells due to damage accumulation or to telomere shortening.

Second, in conditions of moderately elevated stress level, induction of defence systems may prevent further damage accumulation. Thereby stress may be considered as having stimulatory effects on repair systems, as long as these stimulatory effects do not involve the accumulation of irreversible modifications. This is known as hormesis. Pretreatments of *Caenorhabditis elegans* at sublethal temperatures induce significant increases in thermotolerance and small but statistically highly significant increases in life expectancy[2]. However, life span prolongation after transgenesis of flies for antioxidant enzymes[3,4], incubation of cultivated human diploid fibroblasts (HDFs) or *C. elegans* with antioxidant chemicals[5,6], or life span prolongation of $p66^{shc}$ knock-out mice[7] must not be considered as hormetic effects since a longer life span was obtained without extra stress. The possibility also exists that biological systems like cells cultivated *in vitro* at 20% oxygen are not kept in the optimal environmental conditions dictated by millions of years of evolution. These experimental conditions could favor the establishment of some kinds of stress (e.g., oxidative and metabolic) the level of which might be higher than the moderated level of stress allowing hormetic effects. This could explain why the hormetic increase of proliferative life span observed after repeated heat shocks along serial cultures of HDFs under 20% $O_2$ atmosphere[8] is so tiny. It would be worth repeating these experiments at lower $O_2$ tensions, closer to the physiological tensions.

A third situation is chronic stress or (repeated) short stress, for instance, exposures to abnormal ROS concentrations such as produced by inflammation, several types of irradiations, and xenobiotics. In such conditions, the stress response mechanisms specific to a given type of stress might be transiently overwhelmed. If damage is not repaired quickly, there is an increase of the damage level and decrease of the capacity of storing (e.g., ATP and redox potential) and using (biochemical reactions) free energy. This corresponds to the thermodynamic conditions for loss of stability of far-from-equilibrium open systems[1]. A new steady state "found" by the cells, if ever found, would be characterized by two major irreversible differences when compared to the previous one: a higher level of damage and a lower global biochemical activity. Depending on the cell type, these conditions can lead to stress-induced premature senescence-like phenotype (or stress-induced premature senescence, SIPS, depending on the definition of senescence, as discussed later)[9] or trigger apoptotic self-destruction. SIPS or apoptosis will not take place if the level of damage is too high and/or the level of ATP too low. Indeed, a sufficient amount of ATP is necessary for apoptosis to occur (for reviews: [10,11,12]). Protein synthesis, and therefore a sufficient amount of ATP, is necessary for SIPS to occur[13]. As the reader will be reminded below, there also exist activation processes that are able to trigger apoptosis independently of a process, depending on an initial sharp increase in the level of intracellular damage, through the activation of specific signaling processes that launch processes of self-destruction in which a large amount of ROS can be generated, namely, by mitochondria, as one of the final steps of apoptosis.

Independent groups found that the exposure of human proliferative cell types (such as lung and skin fibroblasts, melanocytes, endothelial cells, retinal pigment epithelial cells, and erythroleukemia cells) to subcytotoxic stress (with UV, organic peroxides, $H_2O_2$, ethanol, mitomycin C, hyperoxia, bleomycin, $\gamma$–irradiations, homocysteine, or hydroxyurea) triggers SIPS (for a review [14]). Cells in SIPS share numerous features with senescent cells, such as irreversible growth arrest of the great majority of the cell population and (subsequent) senescence-associated $\beta$-galactosidase (SA $\beta$-gal) activity and stable senescent-like morphology (for a review of possible mechanisms[14]). A recent study attributed SA $\beta$-gal activity to an increase in lysosome size[15].

A fourth situation is cellular activation. On one hand, it is often observed that specific stimulus or stress directly activates signaling pathways that trigger the (over)expression or repression of specific genes that in turn affects the global celular behavior that can adopt trajectories as extreme as apoptosis. On the other hand, there exist activation processes that do not seem to change the global cellular behavior at first sight or after one stimulation. Nevertheless, the border between SIPS and cell activation is not always clear cut. For instance, the signaling pathways activated by tumour necrosis factor-$\alpha$ (TNF-$\alpha$) induce transient intracellular increases in ROS such as $O_2^{(-)}$ and $H_2O_2$ acting as secondary messengers[16,17]. SIPS of HDFs can be induced by repeated stimulation with TNF-$\alpha$ or interleukin-1 (IL-1) added at noncytotoxic and non-pro-proliferative concentration[18]. An increase of the cellular antioxidant potential due to the addition of N-acetylcysteine to the culture medium[19] is highly protective against these changes. TNF-$\alpha$ and IL-1 are examples of proinflammatory cytokines released by cells in response to a wide range of stimuli, such as various microbial products, viruses, immune complexes, activated T cells, and combined action of IL-2 and IFN-$\gamma$[20]. After repeated stimulation by these cytokines, several cell types could undergo SIPS, in addition to the specific tissue effects of these cytokines. Indirect effects of the cellular responses to repeated stimulation by these cytokines on neighbouring cell types are not excluded either, leading to changes in the cellular interactions in the tissues[18]. *In vivo* evidence for such indirect effects of cytokines are listed later.

The fifth type of situation covers cytotoxic injuries: the level of intracellular damage increases and the global biochemical activity decreases so much that the cells die by necrosis[1].

## STRESSES, CELLULAR BEHAVIORS, GROWTH KINETICS, AND CULTURE HETEROGENEITY

From this theoretical framework, one easily understands that a population of cells of a given cell type is never fully homogeneous given the background of each cell considered individually. If cell populations were fully homogeneous, there would be nothing like cytotoxicity curves with dose-dependent decreases of surviving cells; the whole cell populations would die at a given concentration of stressor. The individual background of cells encompasses exposures to different levels of stress(es), asymmetric division process (as proposed for HDFs[21,22,23]), and different numbers of divisions of parent cells due to differential durations of contact inhibition in cell clones. Given this heterogeneity, the response of a cell population to a given condition or stress of calibrated intensity and nature is not an all-or-nothing process. Several cellular behaviors might even be observed in a cell population of a given cell type exposed to such conditions. For instance, both irreversible growth arrest and antiapoptotic effects occur in a culture of HeLa cells overexpressing *c-rel* transcription factor. Overexpression of Mn-superoxide dismutase suppresses those phenotypes[24]. When a cell population is exposed to stress at the subcytotoxic level, it is no surprise that even if a gross majority of cells become irreversibly growth arrested and prematurely “senescent,” a minority of other cells might eventually resume mitosis (Fig. 1).

We have seen above that SIPS of HDFs can be induced by a stimulation repeated five times with TNF-α or IL-1α at noncytotoxic and non-pro-proliferative concentration. Analysis of the data shows that only a proportion of the HDFs undergoes SIPS in those conditions: the increase of the proportion of cells positive for SA β-gal activity does not reach the level observed in senescent cells[18]. It might remain possible that it takes longer for SA β-gal activity to increase in the HDFs after exposures to these cytokines. It is also interesting to note that no cell death was observed after these five stimulations, while cells started apoptosing after a sixth stimulation (unpublished).

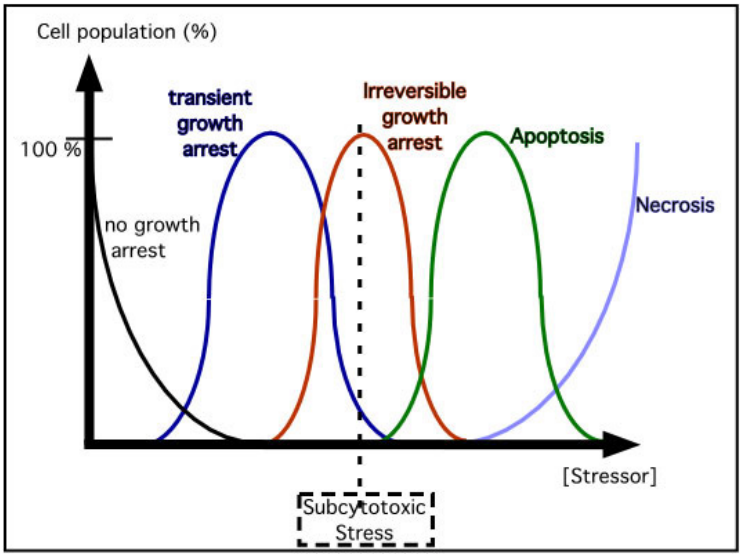

**FIGURE 1.** When a cell population of a given cell type is exposed to stress at subcytotoxic level (vertical dotted line), a gross majority of cells become irreversibly growth arrested and prematurely “senescent” while a minority of other cells might eventually resume mitosis.

Two types of stress protocols are used to increase the proportion of HDFs undergoing "premature senescence" due to stress. These protocols are based on either a continuous chronic mild stress (such as hyperoxia for several weeks) or a repeated discontinuous short subcytotoxic stress (for example, $H_2O_2$ or tert-butylhydroperoxyde, t-BHP). For instance, a recovery of mitosis occurs in a minority of WI-38 HDFs after exposure to 150 µM $H_2O_2$[25], while no recovery is observed after two stresses on IMR-90 HDFs with 75 µM $H_2O_2$[26]. The determination of the stressor dose is crucial in order to remain in subcytotoxic conditions. It would not matter very much if a small proportion of the cell population dies by necrosis or apoptosis, which takes place within 2 days after stress. Indeed, these cells would disappear from the culture before the long-term effects of the stress on "senescence" start being analysed. More important, continuous chronic mild stress and short repeated discontinuous subcytotoxic stress allow the percentage of cells that are only transiently growth arrested to decrease as much as possible. Irreversibly growth-arrested cells in SIPS can be overgrown by the minority of cells recovering growth capacity after stress. As a consequence, along serial population doublings (PDs) after stress, the proportion of cells in SIPS apparently decreases, since they are "diluted." Interestingly, the low proportion of the cell population that recovers mitosis after repeated subcytotoxic stress must divide a greater number of times to reach confluence after subculture. Therefore there will be a decrease of the maximum number of cumulative PDs taking place before the whole cell population becomes growth arrested ("senescent").

## INTERMEDIARY CONCLUSION

In the first part of this article, we described a general theoretical framework to classify various cellular behaviors. Obviously, cellular aging, activation, hormesis, SIPS, apoptosis, and necrosis are different net results of the interactions of the cells with their environment. These net results depend not only on the magnitude (dose and concentration of stressor) and duration of stress but also on the efficiency and kinetics of the defence/repair/elimination systems.

This theoretical framework also helps to conceptualise that a population of cells of a given cell type is never fully homogeneous given the background of the cells considered individually. As far as aging is concerned, this theoretical framework gives a basis for understanding the complex relationships existing between stress and senescence. Slow accumulation of damage allows a *stochastic* counting of time that passes. The long process of damage accumulation is called "damage-dependent senescence" in this article and could explain the gradual senescence of nonproliferative cell types, ending up in apoptosis or not.

In proliferative cell types, telomere shortening occurring discretely at each cell division in the absence of telomerase activity can be seen as a particular example of damage that accumulates due to the lack of an efficient repair system and/or perfect DNA duplication mechanisms and that *discretely* counts cell divisions. The particular effect of telomere shortening on senescence is called "telomere-dependent senescence" herein.

Just before discussing the relative order of occurrence of "telomere-dependent senescence" or "damage-dependent senescence" in proliferative cells and their possible interactions, we must discuss the concept of replicative senescence.

## REPLICATIVE SENESCENCE

In 1961, Hayflick and Moorehead published that serial subcultivations of HDFs exhaust their proliferative potential[27]. HDFs can proliferate vigorously for dozens of generations, but after approximatively 50–70 generations, the cells are growth arrested[28]. This *in vitro* phenomenon has been termed "replicative senescence." More than 80 genes undergo a senescence-related change in relative expression level[29]. The occurrence of replicative senescence has been demonstrated for many cell types. Notable exceptions are embryonic germ cells and the large majority of tumor-derived cells[28,30].

It is unquestionable that telomere shortening is a universal mechanism that limits the proliferative potential of normal human cells lacking endogenous telomerase. Most human primary cells do not express high levels of telomerase and, therefore, are subject to a progressive erosion of their telomeres with each cell generation. The most dramatic consequence of telomere dysfunction is the appearance of chromosomal fusions. Critically short telomeres are probably mistaken for damaged DNA, which results in telomeric fusion. In agreement with this concept, the activation of p53 is the mediator of senescence induced by short telomeres[31,32].

According to the authors, the definition of senescence might be different, and no consensus has been reached so far. The narrowest definition of senescence is the type of irreversible growth arrest triggered by telomere shortening that counts cell generations[33] (definition 1). Other authors enlarged this definition to a functional definition encompassing any kind of irreversible arrest of proliferative cell types induced by damaging agents or cell cycle deregulations caused, for instance, by overexpression of proto-oncogenes[28] (definition 2). According to definition 2, “telomere-dependent” replicative senescence is one of the types of cellular senescence. The aim of this article is not to decide which definition is the most appropriate. Telomere-dependent replicative senescence has also been described as the *ultimate* barrier against cell immortalisation. The exclusivity of telomere-dependent replicative senescence could reside in the word “ultimate.” All other forms of senescence may also represent barriers against immortalisation as long as irreversible growth arrest is observed. Therefore, a common trait between the different forms of senescence would be based on irreversible growth arrest of proliferative cell types and subsequent phenotypic changes. This would exclude the reversible growth arrests due to very mild transient changes in the environment parameters.

The routine practice of cultivating cells under 20% atmospheric oxygen is an obvious source of oxidative stress[28]. In this perspective, considering definition 1 of senescence, any cell cultivated in 20% atmospheric oxygen that irreversibly stops dividing before one of its telomeres reaches a minimal length would be a stress-induced senescence-like phenotype. When all the HDFs of a culture become postmitotic in 20% oxygen, there would be a mosaic made of growth-arrested cells due to accumulation of oxidative damage, on one hand, and cells in telomere-dependent senescence due to discrete telomere shortening, on the other hand. Indeed, a population of cells of a given type is heterogeneous enough so that some of these cells are more rapidly affected by oxidative stress than others and stop dividing earlier than others. When a culture that has been exposed to increased stress during its proliferative life span becomes postmitotic, the proportion of cells in stress-induced premature senescence-like phenotype should be higher than in a culture that has been exposed to lower stress, and the proportion of cells that reached telomere-dependent replicative senescence due to the end-replication problem should be lower (Fig. 2), unless the stress provokes telomere shortening due to DNA damage single-strand breaks, which will be discussed herein.

In conclusion, it might be too simplistic and misleading to consider that the stress response of all the cells of a cell culture (or a tissue) is an all-or-nothing process. One must discriminate between a situation in which all cells of a given population are transiently growth arrested and will resume proliferation and another situation in which a proportion of the cell population remains irreversibly growth arrested while a small proportion of the population will resume mitosis.

Wright and Shay and their colleagues[33,34] proposed it is possible to find culture conditions for each cell type that minimize stress and that leave telomere shortening as the only barrier to immortalisation. When keratinocytes are cultured in chemically defined media, most of the reports found the number of PDs dramatically less than the 50 or so PDs described previously for the growth of keratinocytes on feeder layers[35]. Fifty PDs is more likely to correspond to a situation in which many more cells are in a state of telomere-dependent replicative senescence

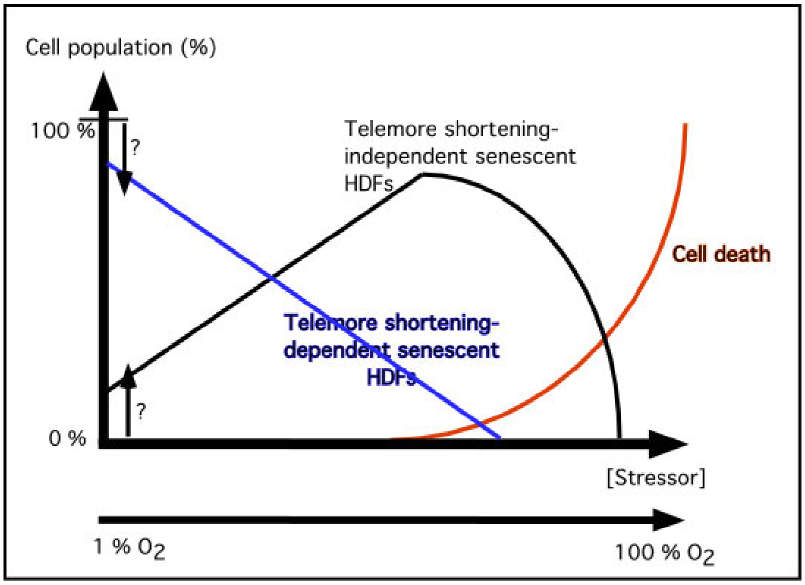

**FIGURE 2.** As stress increases, for instance from 1 to 100% $O_2$, the proportion of cells in stress-induced premature senescence-like phenotype increases, and the proportion of cells which reached telomere-dependent replicative senescence decreases. Cell death increases as stress increases beyond subcytotoxic level. Since oxidative damage accumulates at physiological low $O_2$ partial pressures (damaged DNA bases are found *in vivo* in physiological conditions), a minority of telomere-independent senescent HDFs are likely to appear at low $O_2$ partial pressures.

than the 15–20 PDs observed when a chemically defined medium is used. Human keratinocyte replicative potential without feeder layers is limited by a cyclin-dependent kinase inhibitor (CDKI) $p16^{ink\text{-}4a}$-dependent mechanism, the activation of which can occur independently of telomere length. Abrogation of this mechanism together with telomerase expression immortalizes keratinocytes without affecting other major growth control or differentiation systems[36,37,38].

Using feeder layers increases greatly the number of PDs of human mammary epithelial cells. Cultures of HDFs in chemically defined medium in the presence of 0.25% serum recapitulated a similar scenario and were growth arrested after about 25 PDs regardless of telomere length or telomerase overexpression[33,34]. These cells could be in a state of prolonged GO phase of the cell cycle, since their proliferative capacity can be rescued when they are transferred to adequate culture media with 10% serum (this does not mean that serum cannot have deleterious effects at later stages of culture). Such a state of prolonged GO due to the lack of mitogenic and/or survival serum components is not covered by either of the two definitions of senescence given herein. It is also likely that the time these cells remain in prolonged GO determines whether or not their proliferative capacity can be rescued in a significant portion of the cell population.

In another example of experiments, HDF cultures were left confluent for up to 12 weeks. On resuming cell division, these long-term confluent cultures completed 15–25 fewer PDs than the controls prior to senescence. These lost divisions were mainly accounted for by slow cell turnover of the long-term confluent cultures and by permanent cell cycle exit of 94% of the long-term confluent cells, which resulted in many cell divisions being unmeasured by the PD method (see below for discussion). In the long-term confluent cultures, CDKI $p27^{kip1}$ accumulated and the retinoblastoma protein (Rb) became underphosphorylated and underexpressed. Coincident with permanent cell cycle exit, the long-term confluent cultures upregulated the CDKI $p21^{waf\text{-}1}$ and $p16^{ink\text{-}4a}$. Following subculture of the long-term confluent cultures, the downregulation of $p27^{kip1}$ and the phosphorylation of Rb preceded the complete resumption of normal proliferation rate and downregulation of $p16^{ink\text{-}4a}$. Therefore, HDFs can also accumulate CDKI $p16^{ink\text{-}4a}$, $p21^{waf\text{-}1}$, and $p27^{kip1}$ by senescence-independent reversible mechanisms[39]. In this case as well, it is likely the time these cells remain in prolonged GO will determine whether or not their proliferative capacity can be rescued in a significant portion of the cell population. On the contrary, it would be astonishing to observe that HDFs that have undergone growth arrest at 20% oxygen would resume mitosis when exposed to a decreased oxygen partial pressure.

Survival factors can induce the (over)expression of repair systems, and lack of these factors might favor the accumulation of damage due, for instance, to oxidative stress and apoptosis. If too prolonged, this lack of mitogenic and/or survival factor components might become lethal. If these factors are provided early enough, growth might resume more easily. This concept is not new. For instance, insulin-like growth factor-I extends *in vitro* replicative life span of skeletal muscle satellite cells by enhancing $G_1$/S cell cycle progression via the activation of phosphatidylinositol 3'-kinase/Akt signaling pathway[40].

A high percentage of serum in culture media does not automatically mean that all the cells will reach telomere-dependent replicative senescence. Some components of the serum not only can be oxidized and affect either directly some extracellular matrix or transmembrane effectors or indirectly by triggering oxidative stress by any means but also can produce ROS, for instance, amine oxidase[41]. On the other hand, serum contains antioxidants such as vitamine E or quenchers of ROS such as serum proteins. When HDFs are exposed to cytotoxic concentrations of t-BHP, a much higher survival rate is observed when the same amount of proteins as that assayed in serum is added to the culture medium[42]. Since mouse fibloblasts are much more sensitive to a variety of stress than human fibloblasts[43], it is not very surprising that they acquire a stress-induced premature senescence-like phenotype. In mouse cells, this type of growth arrest seems to be linked with overexpression of CDKI $p16^{ink-4a}$. In HDFs acquiring a stress-induced premature senescence-like phenotype, CDKI $p21^{waf-1}$ remains overexpressed at least for weeks after stress. $p21^{waf-1}$ protein level increases dramatically during the two to three passages preceding senescence (under 20% oxygen), but $p21^{waf-1}$ levels decline once senescence is reached. During this period, $p16^{ink-4a}$ mRNA and cellular protein level gradually rise[44].

Additionally, two recent articles reported that rat oligodendrocyte precursors and Schwann cells[45,46] can be grown indefinitely in appropriate medium with moderate serum conditions.

## TELOMERE SHORTENING AND SIPS: DISCUSSION

Forty percent $O_2$ hyperoxia is mild enough to allow the subcultivation of WI-38 HDFs for several PDs before irreversible growth arrest of the whole population of cells occurs. WI-38 HDFs exposed to 40% $O_2$ undergo a mean TRF (telomeric restriction fragment) shortening of 500 bp/PD in hyperoxia, while normoxic control displays a “normal” shortening of 90 bp/PD. Both hyperoxia-treated and normoxic cells undergo irreversible growth arrest when a mean 5-kb telomere length is reached[47]. In other experiments, the continuous oxidative stress generated by hyperoxia during several PDs was replaced by a stress repeated at every two PDs where WI-38 HDFs were exposed for 1 h to 100 µM t-BHP, allowed to recover for 48 h (stressed cells are sensitive to trypsination and need time to recover before being subcultivated [unpublished]), and plated at 1:4 ratio. When confluence was reached, TRF length measurements were carried out. The control cells proliferated for more than 20 PDs and underwent a 214 ± 48 bp TRF shortening/2 PDs, thus about 107 bp/PD[25], in agreement with previous results[47]. A 490 ± 71 bp TRF shortening/stress, that is, about 245 bp/PD after stress, was found in the cells exposed to t-BHP stress. After 4 stresses for 1 h with 100 µM t-BHP at every two CPD (8 PDs), the cultures became irreversibly growth arrested (Fig. 3). Control and stressed cells irreversibly stopped growing when the mean TRF length was between 4.8 and 5.0 kb[25].

This value of 245 bp/PD is under the 500 bp/PD observed after long-standing mild hyperoxia. This might be due to the specificities of the stress protocols used. On one hand, hyperoxia at 40% is very mild and lasts for weeks during which cells are constantly under hperoxia.

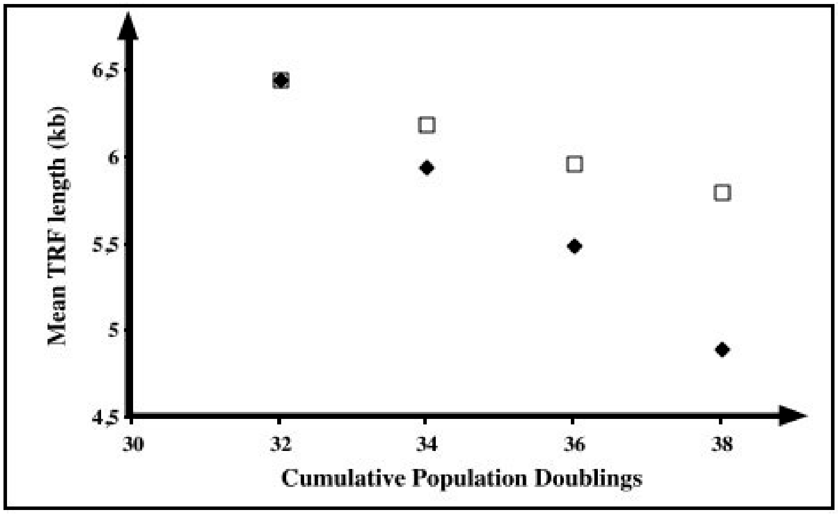

**FIGURE 3.** Effect of repeated t-BHP stress with a stress at every 2 CPDs on telomere shortening in WI-38 HDFs. At every 2 CPDs, the confluent cells were exposed to a subcytotoxic 1-h stress under 100 µM t-BHP. After 48 h of recovery, the cells were plated at 1:4. When confluence was reached, half of the cultures were used for measurement of TRF length while the other half were exposed to a further stress. Four stresses were sufficient to reach growth arrest in the stressed cultures. WI-38 HDFs exposed to t-BHP (♦); Control WI-38 HDFs (open squares).

Cells duplicate their DNA and divide during this hyperoxia. On the other hand, stresses under t-BHP or $H_2O_2$ are short, 1 or 2 h, with subcytotoxic concentrations of the stressors, and the cells are incubated in normal conditions between each stress. These subcytotoxic concentrations of oxidants generate DNA damage immediately after onset of stress, which immediately blocks the cell cycle. The cells that start dividing again at least 2 days after this subcytotoxic stress[48] are most likely those with the lowest level of remaining DNA damage, which explains the lower stress-related TRF shortening observed.

An obvious interpretation is that telomere damage is directly responsible for the decrease in TRF length observed, leading to cell to telomere-dependent growth arrest. A difficulty in this interpretation resides in the idea that if stress-induced DNA damage was responsible for only telomere shortening, a much more important TRF shortening would perhaps be observed. Indeed, oxidative stress generates DNA damage stochastically and probably not only in the last telomeric bp that disappears in hyperoxia or after repeated 1 h t-BHP stress at every two PDs. Another interpretation has been that premature senescence can occur with no significant TRF shortening after oxidative stress. As an apparent argument, it was found that young HDFs exposed to 150 µM $H_2O_2$ once or 75 µM $H_2O_2$ twice in 2 weeks display long-term growth arrest, enlarged morphology, increases in proportion of SA β-gal activity positive cells, and overexpression of apolipoprotein J mRNA. Weekly treatment with 75 µM $H_2O_2$ failed to induce significant TRF shortening. No elevated $p16^{INK4\text{-}a}$ protein or mRNA level was found in $H_2O_2$-treated cells[26]. It is most probable that no telomere shortening occurred in these cells, since telomeric attrition requires cell division[39]. At least this is in favour of an independence of the establishment of premature senescence from telomere shortening. $p16^{INK4\text{-}a}$ protein or mRNA would perhaps increase in premature senescence-inducing protocols in which all $H_2O_2$-treated cells do not undergo premature senescence after stress of milder intensity.

A partial explanation of these results must consider the heterogeneity of the cell population of a given cell type. On one hand, there is a fraction of the cell population that is irreversibly growth arrested after each stress. No TRF shortening occurs in these cells since they are growth arrested. This fraction of irreversibly growth-arrested cells increases with the number of repeated stresses. On the other hand, the fraction of the cell population that recovers mitotic potential after each of the repeated subcytotoxic stress (or that remain mitotic during several weeks of hyperoxia) must divide a greater number of times to reach confluence during the following subculture. Telomere shortening occurs each time these cells divide. The higher number of stress, the smaller fraction of the cell population recovering its mitotic capability and the higher number of times these cells divide (and undergo telomere shortening) to make confluent sheets of cells upon subcultivation.

Michiels et al. found a linear decrease of the growth index with time under hyperoxia[49]. If four culture passages are achieved under 40% oxygen before growth arrest occurs, this linear decrease means that at the middle of passages 1, 2, 3, and 4, respectively, 87.5, 62.5, 37.5, and 12.5% of the cells are still proliferative. The TRF shortening observed in a population consisting of cyclers and noncyclers is the average of both groups, which must be considered for scrupulous estimation of TRF shortening. The data would predict a minimum observable telomere shortening at 4 PD during stress = $[(175 \times 1 \times 90) + (25 \times 1 \times 0)] / (175 + 25) + [(125 \times 1 \times 90) + (75 \times 1 \times 0)] / (125 + 75) + [(75 \times 1 \times 90) + (125 \times 1 \times 0)] / (75 + 125) + [(25 \times 1 \times 90) + (175 \times 1 \times 0)] / (25 + 175) = 179$ bp over 4 PDs, if no DNA damage was involved. Thus, under 40% hyperoxia, compensatory cycling after stress is not sufficient to explain the observed telomere shortening, for at least $(4 \times 500) - 179 = 1821$ bp, which is in favor of telomere shortening due to oxidative damage. When WI-38 HDFs are exposed to stress with 100 μM t-BHP for 1 h at every two CPDs, growth arrest occurs after four stresses (8 PDs). After 4 stresses, the data would predict a minimum observable telomere shortening due to compensatory cycling of 428 bp over 8 PDs. Thus, after these stresses, compensatory cycling after stress is not sufficient to explain the observed telomere shortening, for at least $(8 \times 245) - 428 = 1532$ bp, which is also in favour of telomere shortening due to oxidative damage.

All the biomarkers of senescence studied from 72 h after exposure of HDFs to repeated subcytotoxic t-BHP stress or single subcytotoxic $H_2O_2$ stress are at levels similar to those observed in presenescent HDFs at 80–90% of their proliferative life span. For instance, the proportion of HDFs positive for SA β-gal, with staining performed on nonconfluent cells obtained after stress, is very similar to that observed in presenescent HDFs at 80–90% of their proliferative life span[50]. The proportions of the various morphotypes of HDFs showed that the stressed cultures behaved like presenescent cultures[51,52]. Cells treated with subcytotoxic concentrations of t-BHP and $H_2O_2$ presented a long-term overexpression of the CDKI $p21^{waf-1}$ and Rb hypophosphorylation at levels observed in presenescent cultures[50,53]. Previous studies followed single cells up to 28 days after such stress and estimated that about 1 cell out of 10 resumed mitosis[51]. More precisely, the levels of $[^3H]$-thymidine incorporation gave the estimation that about 16% of cells recover their proliferative capability after repeated t-BHP stress[25]. Let us note there is a difference between the small percentage of cells that recover mitotic capability after repeated t-BHP stress and the small percentage of cells that are still mitotic in presenescent cultures: the telomeres of the first category of cells are still much longer than the telomeres of the second category of cells. Therefore the 16% of cells recovering proliferative capability after repeated t-BHP stress have high proliferative capability. They should undergo a TRF shortening close to that observed after about 3 PDs to reach culture confluence during the first subculture after stress, which is experimentally confirmed[25]. If stress-induced DNA damage was responsible for TRF shortening, a much more important TRF shortening would be observed. Indeed, $H_2O_2$ and t-BHP generate DNA damage stochastically (e.g., not only in these portions of the telomere that will be erased when DNA duplication takes place). In these conditions, it is interesting enough to mention that the TRF shortening observed after these two types of stresses is similar: $322 \pm 55$ bp ($H_2O_2$) and $381 \pm 139$ bp (t-BHP), which is surprising given the stochasticity of DNA damage within specific portions of chromosomic DNA, even if it is known that telomeric DNA is more sensitive to oxidative damage than nontelomeric DNA[54]. $H_2O_2$ and t-BHP have different chemical properties as far as their oxidant potential and preferred molecular targets are concerned, as well as very different hydrophobicities, which sharply modifies reaction kinetics, especially the capability to generate DNA strand breaks. $H_2O_2$ was already used as a potent agent to generate single-strand breaks; it readily crosses cellular membranes and was used at 150 μM for 2 h. t-BHP is hydrophobic, and five stresses were performed at 30 μM for 1 h. Considering that the cells that recover mitotic capacity will divide a little bit more than three times, one reaches a TRF shortening above 194 bp, which leaves little space for significant telomere shortening due to oxidative DNA damage, in these two particular models (322 ± 55 bp [$H_2O_2$] and 381 ± 139 bp [t-BHP]), when considering the limitations of the techniques used for measuring telomere length and the associated standard deviations. This limited space does not totally rule out the long-term existence of DNA single breaks in the low proportion of cells starting to divide again after these stresses, but the chances seem small. Anyway, this confirms that premature senescence can be observed even if the TRF shortening does not reach the minimal size of around 5 kb. TRF shortening is expected to take place when some cells recover their proliferative capability, between days 12 and 18 after $H_2O_2$ stress[25]. This duration might suffice to repair telomere single-strand breaks in a minority of cells. This would explain why no TRF shortening is observed when the stressed cells are further subcultivated after the first PD after stress[25]. Petersen et al.[54] showed that 50% of the $H_2O_2$-induced single-strand breaks are not repaired at day 19 after stress. Due to cell heterogeneity (and inherent stochasticity of aging systems), it is possible that these 50% of single-stranded breaks are mostly present in the cells that did not resume mitosis.

Inhibitors of DNA topoisomerases I and II induced arrest in cell division in HDFs depending on cell divisions. Arrested cells showed a senescence-like morphology and displayed SA β-gal. Accelerated TRF shortening was not observed in the arrested cells while CDKI $p16^{ink4\text{-}a}$ was upregulated. Upon inhibitor removal, the cells resumed growth but their PDs were reduced dose dependently[55]. This dose dependence led these authors to suggest that DNA topoisomerase inhibitors are reversible inducers of premature senescence, which is nonsense according to both definitions of senescence given herein. It seems rather that the higher the concentration of inhibitor, the smaller the fraction of the cell population recovering its mitotic capability when the inhibitor is removed and the higher number of times these cells divide to make confluent sheets of cells upon subcultivation without inhibitor. This would explain why the maximum number of PDs taking place before the whole cell population becomes growth arrested was very much reduced.

## CELL CYCLE, REPLICATIVE SENESCENCE, AND SIPS

It is noteworthy that the p53 pathway is activated when telomeres reach critically minimum length. This activation shares similarities with the activation of p53 due to accumulation of DNA damage[28]. This can be seen as a similarity between telomere-dependent senescence and damage accumulation-dependent senescence, the second type representing a short-cut mechanism favoring irreversible growth arrest.

We have seen above that CDKI $p21^{waf\text{-}1}$ protein expression increases dramatically during the two to three passages before senescence but that $p21^{waf\text{-}1}$ level declines when senescence is reached. During this period, CDKI $p16^{ink\text{-}4a}$ mRNA and cellular protein levels gradually rise, with the protein levels in senescent HDFs reaching nearly 40-fold higher than early passage cells. In senescent HDFs, $p16^{ink\text{-}4a}$ is complexed to both CDK4 and CDK6. Immunodepletion analysis of $p21^{waf\text{-}1}$ and $p16^{ink\text{-}4a}$ from the senescent cell extracts reveals that $p16^{ink\text{-}4a}$ is a major CDKI for both CDK4 and CDK6 kinases[44].

Induction of CDKI $p21^{waf\text{-}1}$ is observed for at least 3 weeks after subcytotoxic stress with $H_2O_2$[53]. $p21^{waf\text{-}1}$ is also overexpressed in HDFs at 72 h after repeated subcytotoxic t-BHP stress. This contradicts Shay and Wright's prediction that $p21^{waf\text{-}1}$ overexpression is reserved for replicatively senescent cells[33]. CDKIs block the phosphorylation of Rb. Hypophosphorylated

Rb is known to inactivate the E2F transcription factor family, which can no longer transactivate the promotor of genes necessary to the S phase of the cell cycle (for a review [56]).

No mitogenic response is observed in $H_2O_2$-induced SIPS after incubation with serum or the usual growth factors[57]. Proto-oncogene *c-fos* mRNA level is sharply diminished in senescent HDFs and in SIPS induced by hyperoxia, mitomycin C, and $H_2O_2$, which can contribute to a decrease in quantity and activity of AP-1 transcription factor in its c-Fos/c-Jun dimeric complex[58,59,60,61]. This decrease in *c-fos* mRNA level represents a similarity between telomere-dependent senescence and damage accumulation-dependent senescence.

The question about the mechanisms of *irreversible* growth arrest in SIPS has not been often studied. Most studies were aimed at finding which molecules responsible for growth arrest are overexpressed (like CDKIs). Very few studies were aimed at finding how these and other markers of growth arrest could be irreversibly maintained. We know that IMR-90 HDFs developing the $H_2O_2$-induced phenotype of SIPS have higher steady-state levels of transforming growth factor-β1 (TGF-β1) mRNA after stress and secrete increased levels of TGF-β1. In addition, stimulation of IMR-90 HDFs with TGF-β1 triggers the appearance of biomarkers of SIPS as different as SA β-gal activity, senescent morphology, and increased mRNA steady-state level of the senescence-associated genes fibronectin, SPARC, apolipoprotein J (apo J), and SM22. Antibodies against TGF-β1 or TGF-β1 receptor II abrogate the overexpression of these genes observed after subcytotoxic $H_2O_2$ stress and the stress-induced appearance of the senescent-like morphology and SA β-gal activity[62]. TGF-β1 induces the release of $H_2O_2$ from IMR-90 HDFs within 8 h following exposure. Diphenyliodonium, an inhibitor of the NADPH oxidase complex of neutrophils and other flavoproteins, inhibits this TGF-β1-induced $H_2O_2$ production[63]. Therefore a constant oxidative stress might be generated once TGF-β1is overexpressed, which constitutes a closed regulatory loop, explaining why cells in SIPS are maintained in a state of irreversible growth arrest.

E2F1 induces a senescent phenotype in HDFs when overexpressed, which stably arrest proliferation and express markers of replicative senescence in response to E2F1. This activity of E2F1 is independent of its Rb binding activity but dependent on its ability to stimulate gene expression. The E2F1 target gene critical for the senescence response appears to be the $p14^{ARF}$ tumor suppressor. Consistent with a critical role for $p14^{arf}$, cells with compromised p53 function are immune to senescence induction by E2F1, as were cells deficient in $p14^{arf}$[64].

Premature senescence can be also induced in HDFs by transfection of oncogenic ras[65]. Ras upregulates PML expression, and overexpression of PML induces senescence in a p53-dependent manner[66]. These findings support the idea that the senescence response is a critical tumor-suppressive mechanism.

A senescence-like growth arrest is also induced in mouse primary embryo fibroblasts by inhibitors of phosphoinositide 3-kinase (PI3K). This growth arrest is correlated with an increase in CDKI $p27^{Kip1}$. Downregulation of other CDKIs as well as other negative cell cycle regulators such as p53 suggest that this growth arrest is less dependent if not independent of these proteins[67]. On the other hand, insulin-like growth factor-I extends *in vitro* replicative life span of skeletal muscle satellite cells by enhancing G1/S cell cycle progression via the activation of phosphatidylinositol 3'-kinase/Akt signaling pathway[40].

Irreversible growth arrest of cells that have been exposed to subcytotoxic oxidative stress and have accumulated DNA damage could be as efficient in avoiding immortalisation as “telomere-dependent” replicative senescence. We shall discuss later the higher possibility of finding cells in stress-induced senescence-like phenotype *in vivo* rather than cells in “telomere-dependent” replicative senescence.

## SIPS AND GENE EXPRESSION

The steady-state mRNA level of many genes is changed similarly in replicative senescence and in hyperoxia-, t-BHP-, and $H_2O_2$-induced SIPS. Among these genes, genes of known functions are fibronectin, osteonectin (SPARC), apo J, $\alpha$1(I)-procollagen, metalloproteinase-1, interferon-$\gamma$, and Mn-superoxide dismutase (SOD2)[50,60].

Apo J is overexpressed in several models of apoptosis. The retrovirus-mediated stable overexpression of apo J increases survival of WI-38 HDFs after exposure to cytotoxic concentrations of t-BHP and ethanol. In addition, it decreases the induction of two biomarkers of SIPS (a senescence-like morphology and SA $\beta$-gal activity) after exposure to subcytotoxic ethanol or t-BHP concentrations. It was concluded that apo J overexpression is protective against apoptotic stimuli (which can be seen as another similarity with telomere-dependent senescence). No effect of apo J overexpression is observed on the proliferative life span of HDFs. Nevertheless, apo J overexpression triggers SPARC overexpression, and SPARC is known to have antiproliferative effects. This apo J-induced SPARC overexpression does not result in an overall inhibition of the proliferative response to several mitogens except platelet-derived growth factor (PDGF)-AB. The selective inhibition of the mitogenicity of PDGF-AB explains why no growth inhibition took place in cells overexpressing apo J cultivated in normal culture conditions with serum, which contains many other growth factors[68].

Stimulation of dermal HDFs with TGF-$\beta$1 results in increased expression of type I collagen and SPARC[69]. TGF-$\beta$1 induces the overexpression of fibronectin and SPARC in human pulp cells[70]. SPARC-null mesangial cells display significantly decreased levels of TGF-$\beta$1 mRNA and secreted TGF-$\beta$1 protein as well as decreased steady-state levels of $\alpha$1(I) procollagen mRNA and protein, compared to cells expressing wild-type SPARC. Addition of recombinant SPARC to SPARC-null cells restores the expression of $\alpha$1(I) procollagen and TGF-$\beta$1 mRNA[71]. Thus, in different systems, TGF-$\beta$1 regulates the expression of fibronectin, SPARC, and $\alpha$1(I)procollagen mRNA, while, in return, SPARC protein levels regulate the expression of TGF-$\beta$1 mRNA, which is very interesting since it is known that TGF-$\beta$1 is overexpressed after subcytotoxic $H_2O_2$ stress[62] and that TGF-$\beta$1 could lead to oxidative stress through TGF-$\beta$1-induced release of $H_2O_2$[63].

## CAN CELLS IN SIPS AFFECT TISSUE AGING?

From definition 1 of senescence, if HDFs can make many more PDs at physiological low oxygen concentrations, this decreases the probabilities of finding “telomere-dependent” replicatively senescent cells *in vivo*. From the two first telomerase-negative cells that appear during *in vivo* differentiation and that will become fibroblasts, $2^{50}$ cells (>$10^{15}$ cells) must be produced before seeing the first telomere-dependent replicatively senescent HDFs after 50 PDs at 20% oxygen. If the cells make 30 more PDs at physiological low oxygen concentrations, this number goes up to $2^{80}$ fibroblasts (>$10^{24}$ cells), which represents several cubic kilometers of cells. Of course, tissular turnover and asymmetric division processes must be considered, which decrease this figure. Long nonproliferative periods *in vivo* in tissues might favor the appearance of single-strand breaks as observed *in vitro* after long periods of confluence[72]. On the other hand, there are many other proliferative cell types in a mammalian organism that should also divide a similar number of times for all of them to become telomere-dependent replicatively senescent, increasing again these volumes of cells. This increases sharply the chances that the irreversibly growth-arrested HDFs found *in vivo* (positive for SA $\beta$-gal activity, let us say) would represent cells in stress-induced premature senescence-like phenotype rather than cells in telomere-dependent replicative senescence.

It seems that cells in SIPS might affect *in vivo* tissue (patho)physiology and aging. HDFs excised from gastric venous ulcers display several features of senescent cells: reduced proliferative capacity, enlarged size, SA β-gal activity, and overexpression of fibronectin. TNF-α is a major component identified in the fluid of these ulcers. When gastric HDFs are exposed to TNF-α, the senescent-like phenotype appears[73,74], which confirms the data obtained *in vitro* on WI-38 HDFs[18,75]. Several reports show that these proinflammatory cytokines induce the degradation of the extracellular matrix[76,77,78]. These data are puzzling, since the overexpression of several metalloproteinases is also observed in senescent HDFs (for a review [29]). It has been observed in HDFs from several types of tissues that exposure to proinflammatory cytokines can trigger the appearance of biomarkers of senescence. Given that these HDFs might participate in the degradation of the extracellular matrix, they are likely to participate in the tissular changes observed in aging. Moreover, human aging is accompanied by an elevation of the circulating levels of TNF-α and IL-1 (for a review [79]). SA β-gal activity positive cells are also found in arteries subjected to balloon angioplasty, chronic hepatitis, tissue surrounding liver carcinomas, and benign prostatic hyperplasia (for a review [28]).

Homocysteine accelerates the rate of cellular senescence through a redox-dependent pathway. This suggests that chronic oxidative stress in the vessel wall may hasten the rate of senescence. The senescent endothelial cells may become proatherogenic, since two surface molecules linked to vascular disease (intracellular adhesion molecule-1 and plasminogen activator inhibitor-1) become overexpressed[80].

## CONCLUSION

The words “inadequate culture conditions”[34] were used to describe any stress-related senescence-like phenotype. This did not consider that stressful situations exist *in vivo* that might lead to such phenotype, especially in situations of repeated stress. Analogously, this is as if brain gerontologists would consider as heretic all the sporadic forms of Alzheimer’s diseases or Parkinson’s diseases. Analogously again, the appearance of a stress-induced senescence-like phenotype, like these age-related neurodegenerative diseases, could be due to exacerbated modifications of a limited number of parameters that also undergo, to a more limited extent, changes related with normal aging among multiple other age-related changes. Exacerbated modifications of this limited number of parameters would then trigger disease-specific signaling pathways.

Common and different pathways are induced after exposure to different kinds of subcytotoxic stress, changing the level of expression of common and different genes. Some of these pathways might share common portions with telomere-dependent replicative senescence. A proteomic study demonstrated that some polypeptides undergo changes in expression level that are either common between senescence and SIPS or specific to senescence or SIPS. The long-term stress-specific changes have been termed “molecular scars”[9,81]. When a critical level of damage is reached, permanent growth arrest takes places. This permanent growth arrest is thought to be induced by mechanisms that share similarities with replicative senescence. Different animal models can be used like superoxide dismutase knockouts lacking SOD2 or SOD1 (Cu-Zn SOD) or senescence-accelerated mice SAM. Abnormal oxidative stress is involved in many inflammatory processes, pathologies, and intoxications. For instance, it is worth examining whether cells taken from inflammatory sites are more prone to SIPS, thereby favouring the “inflamm-aging” theory of aging[82].

Last but not least, research on the mechanisms triggering SIPS is also of primary importance for being able to induce SIPS in cancer cells.

## ACKNOWLEDGMENTS

O. Toussaint is a Research Associate of the FNRS, Belgium. J.-F. Dierick and C. Frippiat have a Fellowship from the FRIA, Belgium. F. Chainiaux is a Research Assistant of the FNRS, Belgium. J.P. Magalhaes thanks FCT, Portugal. V. Royer has a postdoctoral fellowship of the European Union, 5th Framework Programme, Quality of Life, R&D, “Protage” (QLK6-CT-1999-02193). We wish to thank the European Union, 5th Framework Programme, Quality of Life, R&D, “Functionage” (QLK6-CT-2001-00310), and the Région Wallonne, Belgium Initiative Project “Modelage.”
